# A Novel Thioredoxin-Like Protein of *Babesia microti* Involved in Parasite Pathogenicity

**DOI:** 10.3389/fcimb.2022.826818

**Published:** 2022-02-17

**Authors:** Xianyu Piao, Yu Ma, Shuai Liu, Nan Hou, Qijun Chen

**Affiliations:** ^1^ National Health Commission of the People’s Republic of China (NHC) Key Laboratory of Systems Biology of Pathogens, Institute of Pathogen Biology, Chinese Academy of Medical Sciences & Peking Union Medical College, Beijing, China; ^2^ Key Laboratory of Livestock Infectious Diseases in Northeast China, Ministry of Education, Key Laboratory of Zoonosis, College of Animal Science and Veterinary Medicine, Shenyang Agricultural University, Shenyang, China; ^3^ The Research Unit for Pathogenic Mechanisms of Zoonotic Parasites, Chinese Academy of Medical Sciences, Shenyang, China

**Keywords:** *Babesia microti*, immunoprotection, merozoite, pathogenicity, thioredoxin-like protein

## Abstract

Babesiosis poses a serious threat to immunocompromised individuals and the major etiological species of *Babesia* for human babesiosis is *Babesia microti*. Merozoites are a critical stage in the life cycle of *Babesia microti*. Several merozoite proteins have been demonstrated to play important roles in this process; however, most of the merozoite proteins of *B. microti* remain unknown. In the present study, we identified a novel merozoite protein of *B. microti* with similar structure to the thioredoxin (Trx)-like domain of the Trx family, which was named as *B. microti* Trx-like protein (*Bm*TLP). Western blot assays demonstrated that this protein was expressed by *B. microti* during the erythrocytic infection process, and its expression peaked on day 7 post-infection *in vivo*. Immunofluorescence assay further showed that this protein is mainly expressed in *B. microti* merozoites. *Bm*TLP hold both heparin- and erythrocyte-binding properties, which are critical functions of invasion-related proteins. Immunization with recombinant *Bm*TLP imparted significant protection against *B. microti* infection in mice. Taken together, these results suggest that the novel merozoite protein, *Bm*TLP, is an important pathogenic molecule of *B. microti* and may be a possible target for the design of babesiosis control strategy.

## Introduction

Babesiosis is a parasitic disease of the blood caused by *Babesia*, a pathogenic protozoon transmitted between humans and animals by hard ticks. *Babesia* belongs to the phylum Apicomplexa and comprises more than 100 species that infect a wide array of wild and domestic animals, but only few have been shown to infect humans, including *B. microti, B. venatorum, B. bovis, B. divergens, B. duncani, B. bigemina*, and *B. crassa* (Gorenflot et al., 1998; Schnittger et al., 2012). Nonetheless, for the majority of the reported cases of human babesiosis, the major etiological species is *B. microti*.

Babesiosis poses a serious threat to immunocompromised individuals. Asymptomatic, mild, and moderate infections generally occur in individuals who are immunocompetent (Krause et al., 2003; Krause et al., 2008; Vannier et al., 2008; de Ramon et al., 2016), whereas severe infections are common among immunocompromised patients and the elderly (Falagas and Klempner, 1996; Hatcher et al., 2001; Stowell et al., 2007; Krause et al., 2008; Wormser et al., 2010). The fatality rate is up to 21% among those with immunosuppression and 6–9% among hospitalized patients (Hatcher et al., 2001; Krause et al., 2008). Since the first case reported of human infection with *Babesia* in Yugoslavia in 1957 (Skrabalo and Deanovic, 1957), thousands of cases have been reported worldwide (Brasseur and Gorenflot, 1992; Quick et al., 1993; Kim et al., 2007; Gray et al., 2010; Senanayake et al., 2012). Indeed, babesiosis is now considered a global parasitic disease of humans and is attracting significant attention. Hence, there is an urgent need to identify new vaccines for effective control of babesiosis.

During the blood stage of their life cycle, *Babesia* merozoites invade the host erythrocytes, where they develop and multiply. After the divided trophozoites egress from the red blood cell (RBC) by rupturing host cells, the progeny merozoites invade new RBCs again (Yokoyama et al., 2002; Yokoyama et al., 2003; Kumar et al., 2004). Extracellular merozoites are directly exposed in the peripheral blood of hosts and can be eliminated by humoral immunity, whereas antibodies could never reach the parasites inside the RBCs (Yokoyama et al., 2002). Thus, merozoite proteins are potential antigen candidates for babesiosis vaccine and medication development. At present, the research on merozoite proteins of *Babesia mainly focuses on* surface molecules coating the extracellular merozoites, which attach to target erythrocytes and play key roles in erythrocyte invasion, such as merozoite surface antigen (MSA)-1, MSA-2a_1_, MSA-2a_2_, MSA-2b, and MSA-2c in *B. bovis* (Florin-Christensen et al., 2002), surface antigen of *B. microti* merozoites 44 (Wang et al., 2020), *B. microti* surface antigen 1 (Li et al., 2020). However, the detailed molecular interactions between *Babesia* merozoites and the host RBCs and the critical molecules in merozoite development are poorly understood. Identification of novel merozoite molecules is required for the development of preventive measures against babesiosis.

Thioredoxin (Trx) is one of the three major components of thioredoxin system, which has been identified in multiple eukaryotes and prokaryotes to maintain the redox balance (Jortzik and Becker, 2012; Holzerova et al., 2016). Moreover, Trx systems play critical roles in the immune response, virus infection, and cell death *via* interaction with thioredoxin-interacting protein (Lu and Holmgren, 2014). Besides Trxs, Trx-like proteins (TLPs) were also found in many pathogens. TLPs of *Strongyloides ratti and Trichuris suis* were secreted to bind to monocyte and induce release of cytokines to affect the host intestinal mucosa (Ditgen et al., 2016). *Escherichia coli* thioredoxin-like protein YbbN play a role in integrating the activities of different chaperone pathways in *E. coli* and related bacteria (Lin and Wilson, 2011). A novel protein of the thioredoxin (Trx) family, named *Pf*Trx-mero protein, was revealed to localize on merozoites and judged to be an important ligand participating in erythrocyte invasion by *P. falciparum* (Zhang et al., 2013). There are 6 Trx genes of *B. microti* (Huang et al., 2018a). *Bm*Trx2 and *Bm*Trx3 were obtained and investigated. Both of them localize in the cell cytoplasm of *B. microti* merozoites and possess antioxidant enzyme activity (Huang et al., 2018a; Huang et al., 2018b). However, TLP of *B. microti* has not been reported so far. Understanding the molecular characterization and function of *Bm*Trxs and *Bm*TLPs may help to develop Babesiosis vaccines and anti-*Babesia* drugs.

In our study, the sequence alignment of *Pf*Trx-mero protein, which localizes on the surface of *Plasmodium* merozoites, showed a homolog in *B. microti*, named as *B. microti* Trx-like protein (*Bm*TLP). Due to the similar mechanism of host-parasite interaction between the two Apicomplexa parasites *Plasmodium* and *Babesisa*, the Trx-like protein of *Babesia* is likely to be a merozoite protein and play an important role in *Babesia* proliferation. Thus, in the present study, we generated recombinant *Bm*TLP, and further studied its function and expression in *B. microti*. In addition, mice were immunized with recombinant *Bm*TLP, and challenge experiments were performed to evaluate the value of this novel protein as target antigen of immunoprotection for babesiosis.

## Materials and Methods

### Ethics Statement

All animal procedures were conducted in accordance with the animal husbandry guidelines of the Chinese Academy of Medical Sciences and with permission from the Experimental Animal Committee of the Chinese Academy of Medical Sciences with the Ethical Clearance Number BYS20010.

### Parasite and Animals

*B. microti* strain ATCC PRA-99 was obtained from the American Type Culture Collection (Manassas, VA, USA) and stored in liquid nitrogen. Six-week-old male BALB/c mice (special pathogen free) were purchased from Vital River Laboratory Animal Technology Co. (Beijing, China). The parasites were cultured according to standard methods (Lu et al., 2012). Briefly, BALB/c mice were intraperitoneally injected with the immunosuppressant dexamethasone 0.5 mg per mice (18–22g) every day for five consecutive days to suppress immunity. Then, these mice were intraperitoneally inoculated with 200 μL of frozen *B. microti-*infected blood suspension. Smears were prepared with tail blood from mice, and Giemsa staining was performed to monitor the growth and proliferation of *B. microti.* The parasites were used as a stock for the following experiments, 6-week-old male BALB/c mice or immunized mice were intraperitoneally injected with 1 × 10^6^ parasitized erythrocytes. For parasites isolation, the peripheral blood of infected mice was collected and filtered using Plasmodipur (EuroProxima, Amhem, The Netherlands) to remove leukocytes, according to the manufacturer’s instruction. Saponin (10% in phosphate-buffered saline [PBS]) was used to lyse erythrocytes, and the parasite precipitate was collected for the following experiments.

### Sequence Analyses of BmTLP

The gene sequence (https://www.ncbi.nlm.nih.gov/nuccore/XM_021482140.1) and amino acid sequence (https://www.ncbi.nlm.nih.gov/protein/1206245601/) of *Bm*TLP were obtained from the National Center for Biotechnology Information. The signal peptide and transmembrane regions were predicted using Signal P 4.1 Server and TMHMM 2.0, respectively (Petersen et al., 2011). The overall domain structure was then predicted using the CDD protein annotation resource (https://www.ncbi.nlm.nih.gov/Structure/cdd/wrpsb.cgi). The amino acid sequence of *Bm*TLP was aligned with that of *Pf*Trx-like-mero protein using the DNAMAN V6 tool (Lynnon Biosoft, San Ramon, CA, USA). Further alignments were performed with *Bm*TLP, *Pf*Trx-like-mero protein, and other Trx domain-containing proteins from *Homo sapi*ens (NP_001273876.1), *Mus musculus* (AY243534.1), *Drosophila melanogaster* (NP_572212.1), *Arabiodopsis thaliana* (AAF04439.1), and *Schistosoma japonicum* (CAX71381.1) to determine the functional sites of the Trx domain. All these protein sequences were obtained from the National Center for Biotechnology Information.

### Molecular Cloning and Recombinant Proteins Preparation

Total RNA was extracted from *B. microti* parasites using TRIzol reagent (Life Technologies, Waltham, MA, USA) as previously described (Zhang et al., 2016). Genomic DNA was removed from total RNA using the TURBO DNA-free TM Kit (Thermo Fisher Scientific, Waltham, MA, USA), and reverse transcription was performed using SuperScript III Reverse Transcriptase (Thermo Fisher Scientific) according to the manufacturer’s instructions. The gene fragment encoding *Bm*TLP without the 17 signal peptide amino acid at N-terminal was amplified using high fidelity Phusion DNA polymerase (Finnzymes Oy, Espoo, Finland). All primers used in this study (**Supplementary Table 1**) were designed using Primer BLAST (https://www.ncbi.nlm.nih.gov/tools/primer-blast/). The amplified product was purified using a DNA Gel Extraction Kit (Axygen, Corning, NY, USA), cloned into PGEX-4T-1 and PET-28a expression vectors, respectively. The sequence coding for a lysine-to-alanine mutation at the 58 and 353 positions within the two GAG binding motifs of *Bm*TLP was chemically synthesized and cloned into the PGEX-4T-1 vectors. All clones were expressed in *Escherichia coli* BL21 (DE3) (Smith and Johnson, 1988; Flick et al., 2004). The glutathione S-transferase (GST) and His-tagged recombinant proteins were purified using glutathione-Sepharose 4B and His Gravi Trap affinity columns (GE Healthcare, Chicago, IL, USA) respectively, according to the manufacturer’s instructions. All proteins were analyzed by 12% sodium dodecyl-sulfate polyacrylamide gel electrophoresis (SDS-PAGE) and western blot with monoclonal antibodies against His-tag or GST-tag (Cell Signaling Technology, Danvers, MA, USA).

### Transcriptional Analysis of BmTLP-Encoding Gene

*B. microti* parasites were collected at 0, 1, 3, 5, 7, 9, 11, and 13 days post-infection. Total RNA was extracted, and reverse transcription was performed as described above. Real-time quantitative polymerase chain reaction (qPCR) experiments were performed using the SYBR Premix Ex *Taq* II (Takara, Kusatsu, Japan), according to the manufacturer’s instructions, on a 7300 Real-Time PCR System (Applied Biosystems, Waltham, MA, USA). Each reaction was performed in a final volume of 25 μL, containing 12.5 μL of 2 × Brilliant II SYBR green QPCR master mix, 100 ng cDNA, and 1 μL (10 μM) paired primers. The PCR program was performed for 40 cycles with denaturation at 95°C for 30 s, followed by annealing and extension at 60°C for 1 min. Relative expression was analyzed using the SDS 1.4 software (Applied Biosystems). The specific primers for qPCR were designed using Primer BLAST (https://www.ncbi.nlm.nih.gov/tools/primer-blast/) and are listed in **Supplementary Table 1**. *18S* ribosomal RNA of *B. microti* (*Bm*18S) (GenBank ID: M93660.1) was used as reference gene (Huang et al., 2018a). The efficiency of the primer pairs was calculated using the LinRegPCR program based on the raw fluorescence data generated by qPCR (Ruijter et al., 2009).

### Western Blot Analysis of the Recombinant and Native BmTLP

To confirm the expression of *Bm*TLP in the parasites, rabbit polyclonal antibodies were prepared at Beijing Protein Innovation (Beijing, China) by immunizing New Zealand white rabbits with recombinant *Bm*TLP. 10% Saponin (in PBS) was used to lyse erythrocytes, the parasites were washed for three times with PBS and collected by centrifugation. Total protein was extracted from the parasites at the indicated time points (0, 1, 3, 5, 7, 9, 11, and 13 days post-infection) with RIPA buffer (Solarbio Life Science, Beijing, China). Protein concentrations were quantified by using a BCA kit (Pierce Biotechnology, Waltham, MA, USA), according to the manufacturer’s instructions. The extracted protein and recombinant proteins were separated on a 12% SDS-PAGE gel and analyzed by western blot. After electrophoresis, the proteins were transferred to polyvinylidene fluoride membranes (Millipore, Burlington, MA, USA). Rabbit anti-r*Bm*TLP serum (1:1,000 dilutions) was used as primary antibody, and IRDye 800 CW conjugated goat anti-rabbit IgG (H+L) antibody (1:10,000 dilution, Li-COR Biosciences, Lincoln, NE, USA) was used as secondary antibody. In this study, we performed parallel experiments using rabbit glyceraldehyde-3-phosphate dehydrogenase (GAPDH) (Cell Signaling Technology) as the primary antibody (Huang et al., 2018a). Detections were then made using Odyssey (Li-COR Biosciences).

### Detection of BmTLP by Immunofluorescence

To further assess the location of the *Bm*Trx-like protein in the parasite, immunofluorescence assays were performed. Blood smears were prepared with tail blood from *B. microti*-infected mice at day 7 post-infection. After fixing with methanol for 10 min at 4°C, 0.1% TritonX-100 was used to penetrate. Then the slides were incubated with 5% bovine serum albumin for 1 h and then washed three times with PBS. Rabbit anti-BmTLP serum (diluted 1:500 in 3% bovine serum albumin) was applied as primary antibody at 4°C overnight. The slides were then incubated with Alexa-Fluor 488 conjugated goat anti-rabbit IgG antibody and 4ʹ, 6-diamidino-2-phenylindole (DAPI) (Invitrogen, Waltham, MA, USA). The samples were examined using a confocal laser-scanning microscope (Zeiss LSM 880, Oberkochen, Germany).

### Heparin-Binding Assay With Recombinant Proteins

The binding of BmTLP to heparin was studied as previously described with some improvement (Chen et al., 1998). In briefly, BmTLP (final concentration 0.1 μg/μl) and equal amounts of GST protein were mixed with 100 µL heparin-sepharose or uncoupled sepharose 4B (GE Healthcare) in a 200 µL reaction system in PBS at 25°C for 2 *h*. Binding proteins were eluted by using 1 M sodium chloride buffer and detected by western blot using monoclonal antibodies to the GST-tag.

### Binding Assay of Recombinant Proteins to Human and Mouse Erythrocytes

The binding activity of *Bm*TLP toward human and mouse erythrocytes was studied as previously described with some improvements (Wang et al., 2018). In briefly, *Bm*TLP (final concentration 0.1 μg/μl) and equal amount of GST protein were mixed with 100 µL human or mouse erythrocyte in a total of 200 µL system in RBC binding buffer (50 mM Tris HCl, 200 mM NaCl, 1 mM EDTA, 2.5 mM MgCl_2_, 2.0 mM DTT, 1% glycerin, pH 8.0), respectively, at 25°C for 2 h. The mixture was washed three times with RBC binding buffer. Binding proteins were subsequently eluted with 1 M sodium chloride. The bound proteins were then detected by western blot using monoclonal antibodies to GST-tag.

### Immunization and Challenge Experiments

To assess the protective role of *Bm*TLP immunization against babesiosis infection, immunization was performed with His-tagged recombinant *Bm*TLP and PBS as control. Mice were evenly divided into immunization and control groups. In the first immunization, each mouse (6-week-old male BALB/c mice) in the immunization group was subcutaneously injected with 60 µg His-tagged *Bm*TLP or corresponding volume of PBS emulsified with complete Freund’s adjuvant. In the three subsequent immunizations, each mouse was injected with 30 µg recombinant proteins or corresponding volume of PBS with incomplete Freund’s adjuvant every 2 weeks. The antibody titer was measured using enzyme-linked immunosorbent assay (ELISA) as previously described (Wang et al., 2018). After successful immunization, mice were challenged with 1 × 10^6^ infected (i)RBCs. Thin blood smears were made with tail blood from mice and stained with Giemsa to assess parasitemia every other day by counting 1,000 RBCs per smear and totally three smears as previously described (Wang et al., 2018). The interferon (IFN)-γ and tumor necrosis factor (TNF)-α levels in mouse serum were determined using Mouse IFN-γ ELISA Kit and Mouse TNF-α ELISA Kit (R&D Systems, Minneapolis, MN, USA), according to the manufacturer’s instructions.

### Statistical Analysis

The data were analyzed using GraphPad Prism 5.0 (GraphPad Software, San Diego, CA, USA) and Microsoft Excel 2010. The statistical significance of experimental data was evaluated using Mann-Whitney test between two groups and one-way analysis of variance (ANOVA) among more groups. Values of *p* < 0.05 were deemed significant differences.

## Results

### A Novel Thioredoxin-Like Protein of Babesia

**Figure 1:**
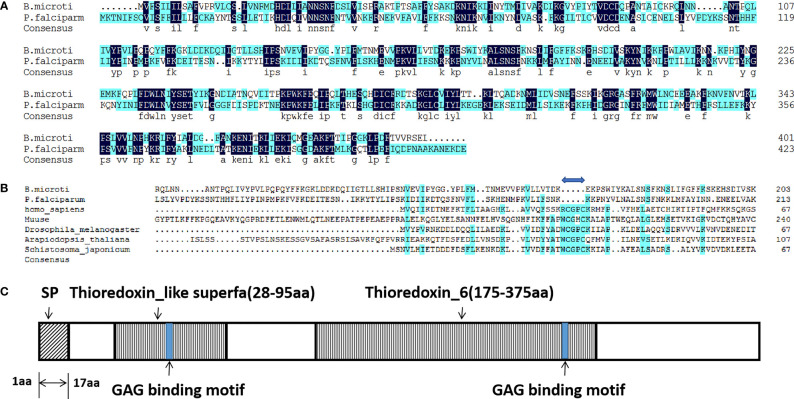
Amino acid sequence alignments and schematic diagram of *Bm*Trx-like protein. **(A)** Amino acid sequence alignment of Trx-like proteins of *B. microti* and *P. falciparum*. **(B)** Amino acid sequence alignment of the Trx-like protein of *B. microti* with Trx proteins of other species. A gap (blue double-headed arrow) was observed in both *Bm*Trx-like protein and *Pf*Trx-like-mero protein sequences due to the deletion of the Cys-X-X-Cys (CXXC) active site. **(C)** Schematic diagram of the *Bm*Trx-like protein. This protein contained a signal peptide (SP; aa 1–17) at the *N-*terminus and two conserved Trx domains (Thioredoxin_like superfa, aa 28–95; Thioredoxin_6, aa 175–375). The blue boxes indicate the two glycosaminoglycan (GAG)-binding motifs.

In our earlier study on heparin-binding merozoite proteins of *P. falciparum*, a novel protein, *Pf*Trx-mero protein, containing a conserved domain of *Pf*Trx-like-mero was identified (Zhang et al., 2013; Wang et al., 2018). In the present study, we report a novel protein encoded by BMR1_03g03170 in *B. microti*, which was homologous to *Pf*Trx-like-mero of *P. falciparum* (Identities: 36%, **Figure 1A**). The amino acid sequence of this *Babesia* protein was found to contain two Trx-like domains (**Figure 1C**); thus, it was named as *B. microti Bm*Trx-like protein (*Bm*TLP). The Cys-X-X-Cys (CXXC) active site, which is essential for antioxidant function, was identified within the sequence of most of the Trx-like proteins. However, the ‘-CXXC-**’** motif was missing in the sequence of both, *Pf*Trx-mero and *Bm*Trx-like proteins (**Figure 1B**). The sequence of *Bm*TLP contained a 17-amino acid long signal peptide (**Figure 1C**), indicating that the protein may have a role on the surface or in the endoplasmic reticulum of the parasites, or even outside of the organism.

### Recombinant BmTLP Can be Identified by Anti-Babesia Mice Serum

**Figure 2:**
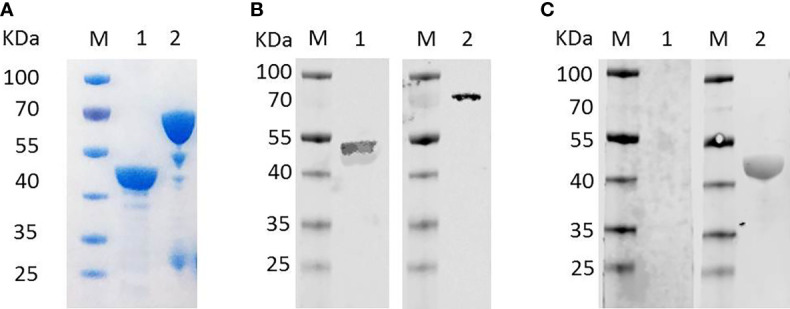
Detection of recombinant *Bm*Trx-like protein (*Bm*TLP). **(A)** Recombinant *Bm*TLP were detected by SDS-PAGE and Coomassie brilliant blue staining. Lane 1, His-tagged r*Bm*TLP; Lane 2, GST-tagged r*Bm*TLP. **(B)** His-tagged (lane 1) and GST-tagged (lane 2) recombinant *Bm*TLP were detected by western blot with monoclonal antibodies against His-tag (Lane 1) or GST-tag (Lane 2) respectively. **(C)** His-tagged recombinant *Bm*TLP were detected by western blot with a mixture of serum samples (equal volumes) from seven normal mice (Lane 1) or seven *B. microti*-infected BALB/c mice 21-day post-infection (Lane 2). Lane M, standard protein molecular weight marker.

Recombinant *Bm*TLP (r*Bm*TLP) with a His-tag or a GST-tag was generated. The molecular weight was 46 kDa for the recombinant His-tagged *Bm*TLP and 72 kDa for the GST-tagged protein (**Figure 2A, B**). Anti-*B. microti* serum, obtained from mice 21 days post-infection of *B. microti*, was used to detect the r*Bm*TLP by western blot. As shown in the western blot analyses, recombinant r*Bm*TLP could be identified by infected serum but not by serum from uninfected mice (**Figure 2C**).

### BmTLP Were Mainly Expressed in *B. microti* Merozoites

**Figure 3:**
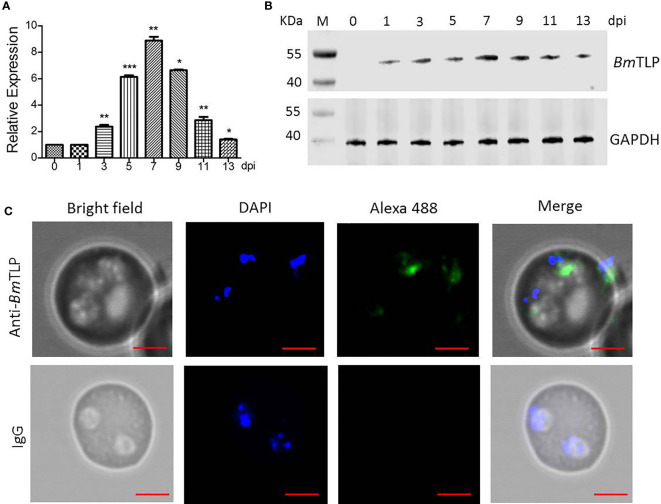
Expression of Trx-like protein (*Bm*TLP) *in B. microti. B. microti* parasites were collected from *Babesia*-infected mice 0, 1, 3, 5, 7, 9, 11, and 13 days post-infection (dpi). **(A)** Transcriptional expression of *Bm*TLP-encoding gene in *B. microti* parasites from infected mice detected by real-time qPCR with *18S* ribosomal RNA of *B. microti* used as reference gene. The results are representative of three independent experiments, with data indicating mean + standard deviation. Asterisks indicate comparison with the previous group with ^*^ *p* < 0.05, ^**^ *p* < 0.01, and ^***^ *p* < 0.0001. **(B)** Expression of *Bm*TLP detected by western blot. Lane M, standard protein molecular weight marker. Lysates of parasite were detected with rabbit anti- *Bm*TLP serum. Glyceraldehyde-3-phosphate dehydrogenase (GAPDH) was used as internal reference. **(C)** Localization of *Bm*TLP detected by immunofluorescence assay. Thin blood smears were prepared with tail blood from *B. microti-*infected BABL/c mice at day 7 post-infection. Proteins were detected with rabbit anti-r*Bm*TLP serum or rabbit IgG control, followed by anti-rabbit IgG secondary antibody conjugated with Alexa-488 (green). Nuclei were stained with DAPI (blue). Scale bar, 2 µm.

Mice were infected with *B. microti* to obtain parasites at indicated times, the dynamic parasitemia of the mice were showed in **Supplementary Figure 2**. The expression features of *Bm*TLP were further explored. Transcriptional expression of *Bm*TLP-encoding gene was detected by real-time qPCR at 1, 3, 5, 7, 9, 11, and 13 days post-infection. Both the primers of *Bm*TLP and *Bm*18S showed high amplification efficiency (96.7% for *Bm*TLP and 91.1% for *Bm*18S). Specific gene expression increased on day 3 post-infection, peaked on day 7, and then gradually decreased (**Figure 3A**). Rabbit anti-*Bm*TLP serum was used to detect native protein in the parasites by western blot. Specific bands could be detected in parasite lysates since day 1 post-infection, peaked at day 7 post-infection, and a sharp decline of the expression was observed on day 13 post-infection (**Figure 3B**). The location of *Bm*TLP was detected by indirect immunofluorescence, with DAPI showing the nuclear chromatin (**Figure 3C**). *Bm*TLP may locate in the cytoplasm of merozoites. There was no positive fluorescence of *Bm*TLP in infected erythrocytes incubated with normal rabbit IgG. Results indicated that *Bm*TLP is expressed in merozoites of *B. microti*.

### BmTLP Adhered to Heparin and Host Erythrocytes

**Figure 4:**
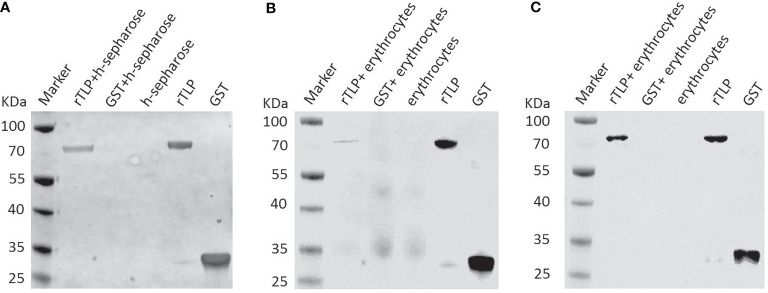
Binding activity of recombinant *Bm*Trx-like protein (*Bm*TLP). The glutathione S-transferase (GST) tagged r*Bm*TLP (rTLP) and GST protein (GST) were incubated with **(A)** heparin-sepharose (h-sepharose), **(B)** mouse erythrocytes, or **(C)** human erythrocytes at 25°C for 2 h Recombinant proteins was incubated with sepharose without heparin as negative control. Eluted proteins were collected and detected by western blot. The GST tagged r*Bm*TLP and GST were used as positive controls. Rabbit anti-GST Tag monoclonal antibody was used as primary antibody. The results are representative of three independent experiments.

To elucidate the role of the *Bm*TLP in the invasion process, GST-tagged recombinant proteins were separately incubated with heparin-sepharose, and the binding proteins were then detected by western blot. As shown in **Figure 4A**, GST-tagged r*Bm*TLP could bind heparin, whereas the GST protein did not. Further assays were performed to detect the binding activity of the *Bm*TLP to the host erythrocytes. The results showed that *Bm*TLP can bind both human **(** **Figure 4B** **)** and mouse erythrocytes **(** **Figure 4C** **)**. To further investigate whether the binding function of *Bm*TLP plays a role through the GAG binding motifs, we introduced a lysine-to-alanine mutation at the 58 and 353 positions within the two GAG binding motifs of *Bm*TLP. The mutant r*Bm*TLP showed no binding activity (**Supplementary Figure 1**).

### Immunization With BmTLP Protected Mice Against *B. microti* Infection

**Figure 5:**
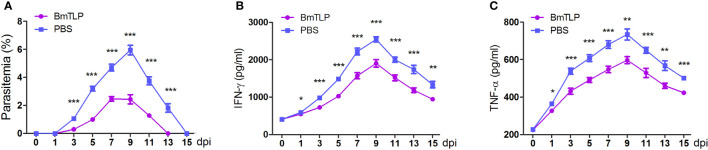
Immunization with recombinant *Bm*Trx-like protein (*Bm*TLP) protected mice against *B. microti* infection. His-tagged *Bm*TLP or PBS was used to immunize BALB/C mice (seven mice per group). After successful immunization, a challenge with 1 × 10^6^ *B. microti-*infected red blood cells was implemented. Parasitemia **(A)** was examined with tail blood by performing Giemsa staining and IFN-γ **(B)** and TNF-α **(C)** in mice serum were detected by ELISA 0, 1, 3, 5, 7, 9, 11, 13, and 15 days post-infection (dpi). The results are representative of two independent experiments. Data indicate the mean ± standard deviation. Asterisks indicate comparison between the two groups at each time point with ^*^ *p* < 0.05, ^**^ *p* < 0.01, and ^***^ *p* < 0.0001.

Immunization with *Pf*Trx-mero protein has been proven to provide significant protection against *Plasmodium* infection (Wang et al., 2018); thus, we further studied the protective role of *Bm*TLP immunization against *B. microti* infection *in vivo*. His-tagged *Bm*TLP was used to immunize BALB/C mice. The titers of antibodies in mice serum were detected by ELISA after the third booster immunization (**Supplementary Figure 3B**). After successful immunization confirmed by ELISA, a challenge with 1 × 10^6^ *B. microti-*iRBCs was implemented. The parasitemia was significantly lower in the *Bm*TLP immunized groups; especially at day 9 post-infection, the parasitemia decreased more than 50%, compared to that in the PBS control group (*Bm*TLP group *vs.* PBS group, 2.43% ± 0.33% *vs.* 5.95% ± 0.30%, *p* < 0.0001, **Figure 5A**). Although there was no significant difference in the body weight of mice between the two groups (**Supplementary Figure 4A**), the spleen weight of the *Bm*TLP immunized mice was significantly lower than that of the PBS control (**Supplementary Figure 4B**). Furthermore, the levels of the inflammatory cytokines IFN-γ and TNF-α in *Bm*TLP immunized mice were much lower than those in PBS control mice (**Figures 5B, C**), indicating that r*Bm*TLP immunization may inhibit the excessive inflammatory response induced by Babesia infection. These results suggest that *Bm*TLP immunization reduces erythrocyte invasion of the parasites and inflammation level of the host under the *B. microti* infection.

## Discussion

Babesiosis imposes an enormous threat on immunocompromised individuals, with death from babesiosis occurs in up to 20% individuals within this group (Hatcher et al., 2001; Krause et al., 2008). The incidence of *B. microti* infections has increased and the geographic range has expanded globally in the past two decades (Gorenflot et al., 1998; Vannier and Krause, 2012). Treatment of babesiosis generally depends on antibiotics, such as atovaquone and azithromycin (Krause et al., 2000; Kletsova et al., 2017). However, antibiotic therapy may not adequately treat immunocompromised patients (Gorenflot et al., 1998). A potent vaccine based on microbial recombinant antigens would be the most effective tool to prevent this disease. Effective *B. bovis* and *B. bigemina* vaccines have been developed for use in cattle (Brown and Palmer, 1999), and a *B. rossi* vaccine has been used for dogs (Schetters et al., 2007), but no human *Babesia* vaccine has been developed to date. Progress has been limited due to the small quantity of identified parasite proteins in human *Babesia*. Thus, exploration of novel proteins that play critical roles in the development stages of *Babesia* will provide candidates for human babesiosis vaccine development.

In the erythrocytic phase of human *Babesia*, merozoite is the only stage that directly contacts with the host peripheral blood (Gorenflot et al., 1998; Vannier and Krause, 2012). The other stages all occur within erythrocytes. Erythrocytes have only basic metabolic activity and no antigen-presenting pathways; therefore, parasitism of erythrocytes provides *Babesia* with an evolutionary advantage in evading host recognition (Gorenflot et al., 1998). Furthermore, merozoite proteins play key roles in erythrocyte invasion by adherence to and penetration into erythrocytes (Baum et al., 2009). Thus, the proteins of merozoite are optimal candidate antigens for a Babesiosis vaccine. In the present study, a novel *B.microti* Trx-like protein *Bm*TLP was identified and found to localize in the cell cytoplasm of *B. microti* merozoites.

The amino acid sequence of *Bm*TLP contains two Trx-like domains. Trxs are disulfide oxidoreductases that utilize a pair of cysteines in the active site, Cys-X-X-Cys (CXXC), to reduce disulfide bonds (Ju et al., 2015; Wedmann et al., 2016; Peng et al., 2017). The active site cysteines of mammalian thioredoxin were reported to be important for the reduction of a protein persulfide since mutation of the attacking cysteine of Trx weakens the binding affinity to persulfidated GAPDH (Ju et al., 2015). However, the CXXC motif was missing in the sequence of both *Pf*Trx-mero and *Bm*Trx-like proteins. The Trx domain of *Escherichia coli* Trx-like protein YbbN also lacks a canonical CXXC active site architecture and YbbN is not a functional oxidoreductase, but it can regulate the activities of chaperones (Lin and Wilson, 2011). *Pf*Trx-mero protein was judged to be an important ligand participating in erythrocyte invasion by *P. falciparum* (Zhang et al., 2013). Thus, *Bm*TLP may be primarily useful for *B. microti* in other ways than antioxidant.

Trxs, despite its function as an intracellular disulfide reducing enzyme, has been found to play some extracellular roles. Human Trx was first identified as a secreted protein of the adult T cell leukemia and implicated in a wide variety of cell-to-cell communication systems, acting as a cytokine or a chemokine (Tagaya et al., 1989). The same extracellular Trx was identified in the conditioned medium of Epstein-Barr virus-transformed lymphocytes and acts as an autocrine growth factor and synergizes with interleukin 1 and interleukin 2 (Wakasugi et al., 1990). However, *Bm*TLP is localized in the cell cytoplasm of merozoites in *B. microti*-infected erythrocytes. Unlike mammalian Trx that are lack of signal sequences, a classical signal peptide sequence was predicted at the *N*-terminus of both *Pf*Trx-like-mero protein and *Bm*TLP. Even in the absence of signal peptides, Trxs can be secreted through unconventional protein secretion pathways, but not the endoplasmic reticulum and Golgi pathway (Rubartelli et al., 1992). These data strongly suggests that *Bm*TLP may be secreted extracellular.

The biological functions of extracellular thioredoxin have also been proved to be involved in the immune response with or without thiol-oxidoreductase activity. Trx is secreted by CD4^+^ T cells and can reduce the disulfide in domain 2 of CD4, which are important for the entry of HIV-I into susceptible cells (Matthias et al., 2002). Trx is shown to be a growth promoting factor for several lymphoid cells with synergistic effects with cytokines such as IL-1 and IL-2 (Tagaya et al., 1990; Wakasugi et al., 1990). TRX80, a proteolytic product of Trx 1, were recruited by the immune system to exert cell communication functions, such as promoting proinflammatory macrophage phenotype (Mahmood et al., 2013) and activating the classical and alternative pathways of complement activation (King et al., 2012). The thioredoxin-like protein can down-modulate the innate immune responses by inhibiting ASK1-MAPKs signaling cascades during *Edwardsiella piscicida* infection (Yang et al., 2019). Trx-like protein from gastrointestinal parasitic nematodes *Strongyloides ratti* and *Trichuris suis* is secreted and bind to monocytic and intestinal epithelial cells and induce the time-dependent release of cytokines such as TNF-α, IL-22, and TSLP (Ditgen et al., 2016). Our *in vivo* experiments showed that r*Bm*TLP immunization could reduce the production of inflammatory cytokines IFN-γ and TNF-α in host circulation, thus may inhibit the excessive inflammatory response induced by *Babesia* infection. However, the immunoregulatory function of Trx is quite complicated and needs further study.

*Bm*TLP contains two Trx domains and two glycosaminoglycans-binding motifs, which are both believed to be related to invasion or adhesion of the parasite (Wang et al., 2018). *In vitro* experiments showed that recombinant *Bm*TLP could bind to host erythrocytes and had heparin-binding activity. Heparin-binding molecules on the surface of merozoites play a crucial role in the erythrocyte invasion process (Bork et al., 2004). Heparin sulfate-like moieties on the surface of human erythrocytes have been shown to be receptors for merozoite-derived proteins of *Plasmodium falciparum* (Vogt et al., 2004; Boyle et al., 2010). *Toxoplasma gondii* surface antigen also has heparin-binding properties and mediates attachment of the tachyzoite to the cellular heparin sulfate proteoglycans of host cells (Jacquet et al., 2001). These data suggested *Bm*TLP may be involved in merozoite-erythrocyte communication. However, whether and how *Bm*TLP could be secreted by *B. microti* needs further study.

Finally, *in vivo* assays were performed to demonstrate the role of the *Bm*TLP in parasite development and proliferation. The r*Bm*TLP immunized mice showed much lower parasitemia during infection and early clearance of the parasites. In line with the parasitemia, the serum level of inflammatory cytokines was much lower in the r*Bm*TLP immunized mice, which may lead to milder symptoms during disease progression, as inflammation induced by the parasites is the main cause of clinical symptoms in protozoan infection (Schofield and Grau, 2005). Thus, immunization with *Bm*TLP resulted in accelerated clearance of the parasite during the infection with lower inflammatory levels. These data indicate that *Bm*TLP plays critical roles in *Babesia* infection. However, until now, the *in vitro* cultivated system and gene manipulating methods of *B. microti* are not mature and it is difficult to confirm the direct involvement of *Bm*TLP in parasite invasion *in vitro*. Recently, two studies reported the establishment of transient or stable transfection method in *B. microti* and the discovery of a novel bidirectional promoter of *B. microti* (Jaijyan et al., 2020; Liu et al., 2020), raising hope for gene-editing research in *Babesia*.

In summary, we have identified a novel Trx-like protein in *B. microti*, named as *Bm*TLP, which mainly expressed in *Babesia* merozoites and holds heparin and erythrocyte-binding properties. Immunization with r*Bm*TLP provided significant protection against *B. microti* infection *in vivo*, thereby suggesting the important roles of this protein in parasite pathogenicity and its potential value for the development of babesiosis vaccines. However, the exact pathogenic mechanism of *Bm*TLP still needs to be investigated.

## Data Availability Statement

The original contributions presented in the study are included in the article/**Supplementary Material**. Further inquiries can be directed to the corresponding authors.

## Ethics Statement

The animal study was reviewed and approved by the Experimental Animal Committee of the Chinese Academy of Medical Sciences.

## Author Contributions

NH and QC conceived and designed experiments. XP and YM performed the majority of the experiments. SL performed some experiments. NH, XP, and QC analyzed the data and wrote the manuscript. All authors reviewed the results and approved the final version of the manuscript.

## Funding

This research was funded by the CAMS Innovation Fund for Medical Sciences (CIFMS) (grant numbers 2021-I2M-1-038 and 2019-I2M-5-042), the National Natural Science Foundation of China (grant number 81672050).

## Conflict of Interest

The authors declare that the research was conducted in the absence of any commercial or financial relationships that could be construed as a potential conflict of interest.

## Publisher’s Note

All claims expressed in this article are solely those of the authors and do not necessarily represent those of their affiliated organizations, or those of the publisher, the editors and the reviewers. Any product that may be evaluated in this article, or claim that may be made by its manufacturer, is not guaranteed or endorsed by the publisher.
