# Supplementary material for: A Novel Thioredoxin-Like Protein of Babesia microti Involved in Parasite Pathogenicity
Source: Front Cell Infect Microbiol. 2022 Feb 17;12:826818. doi: 10.3389/fcimb.2022.826818 (PMC8892138; doi:10.3389/fcimb.2022.826818)
Supplement: Supplementary file 1 [file DataSheet_1.docx]

**Supplementary Figures and Tables**

**Supplementary Table 1.** List of primers used in the study

| **Primer name** | **Primer sequence** | |
| --- | --- | --- |
| *Bm*TLP-F for clone | GGATCC TCGCTG GTGAATAGGA TGGATC | |
| *Bm*TLP-R for clone | CTCGAG CTATAG TTCAGAGCGC ACGAC | |
| *Bm*TLP-F for qPCR | | CCCTAGGGCTAAAACGCCAA |
| *Bm*TLP-R for qPCR | TTGCAGTGTTTGCAGGTTGA | |
| *Bm*18S-F for qPCR | GTTATAGTTTATTTGATGTTCGTTT | |
| *Bm*18S-R for qPCR | AAGCCATGCGATTCGCTAAT | |

Abbreviations: *Bm*TLP, *Babesia microti* thioredoxin-like protein; *Bm*18S, *Babesia m*icroti 18S ribosomal RNA; qPCR, quantitative polymerase chain reaction; F, forward primer; R, reverse primer.

**
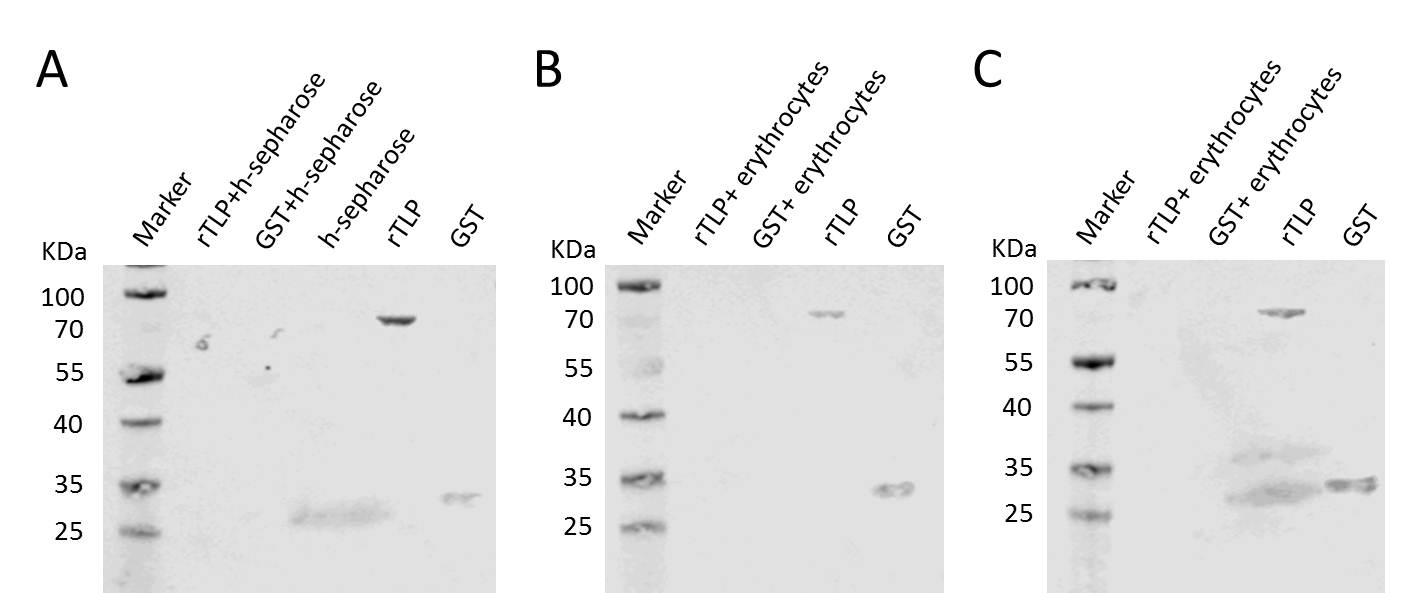
**

**Supplementary Figure 1.** Binding activity of mutant r*Bm*Trx-like protein (*Bm*TLP). A lysine-to-alanine mutation was introduced at the 58 and 353 positions within the two GAG binding motifs of *Bm*TLP (mTLP). The glutathione S-transferase (GST) tagged mTLP and GST protein (GST) were incubated with (A) heparin-sepharose (h-sepharose), (B) mouse erythrocytes, or (C) human erythrocytes at 25°C for 2 h. Recombinant proteins was incubated with sepharose without heparin as negative control. Eluted proteins were collected and detected by western blot. The GST tagged mTLP and GST were used as positive controls. Rabbit anti-GST Tag monoclonal antibody was used as primary antibody.

**
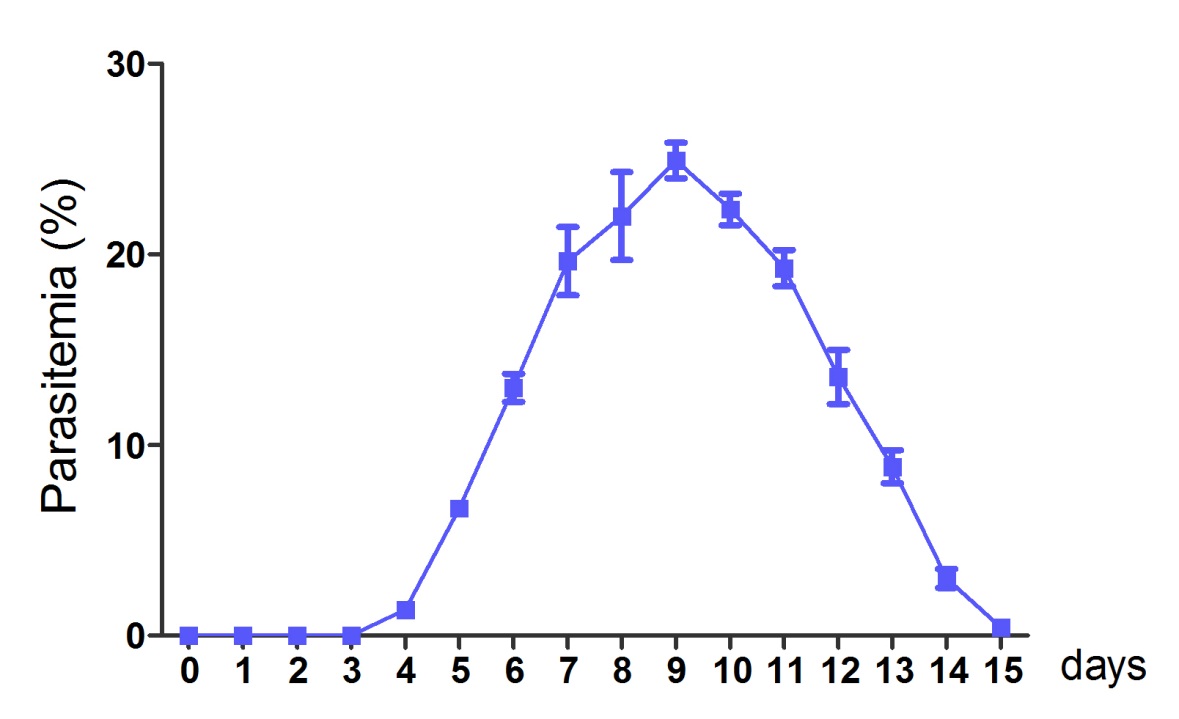
**

**Supplementary Figure 2.** Dynamic parasitemia of *B. microti* infected mice. Six-week-old BALB/c mice were infected with 1 × 106 parasitized erythrocytes, parasitemia was examined with tail blood by performing Giemsa staining everyday post infection.

**
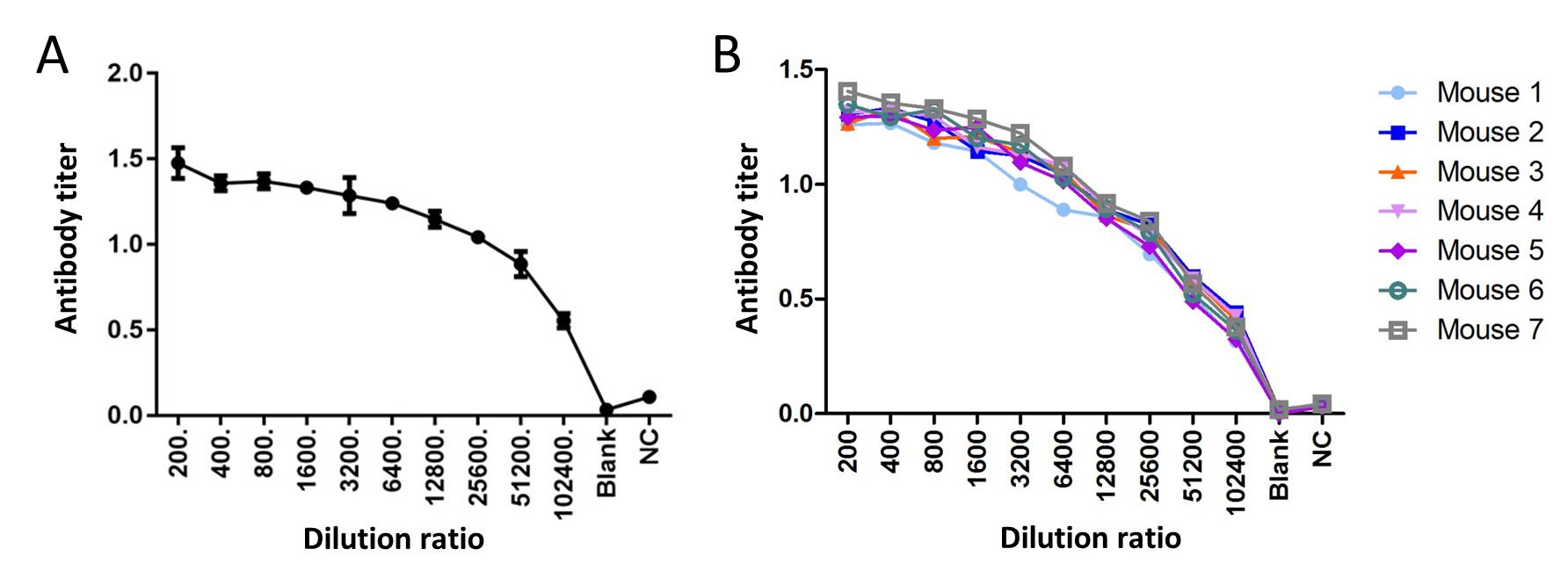
**

**Supplementary Figure 3.** The titer of rabbit and mice antibodies. New Zealand white rabbit and BALB/c mice were immunized with His-tagged *Babesia microti* thioredoxin-like protein. The titers of antibodies in the serum of rabbit (A) or mice (B) were detected by ELISA after the third booster immunization.

**
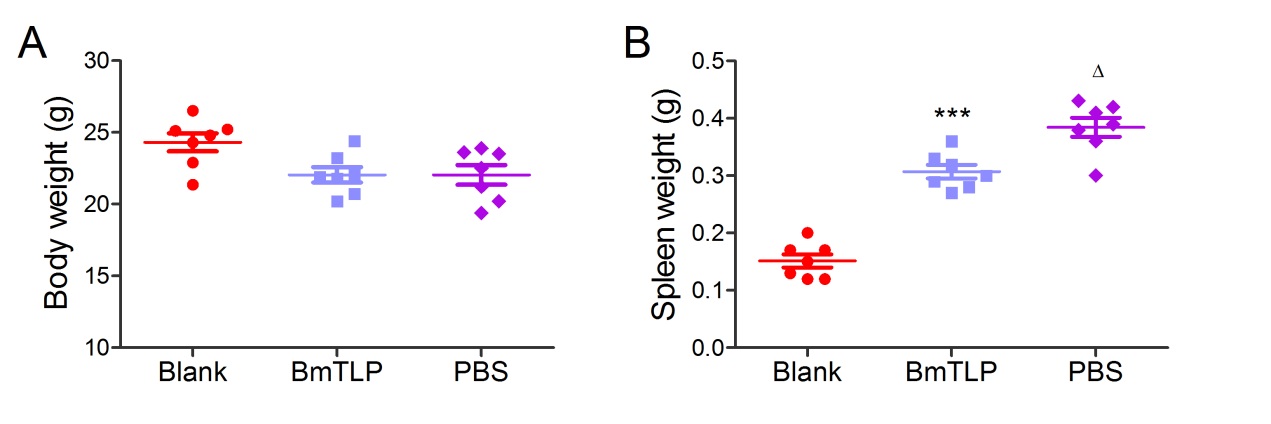
**

**Supplementary Figure 4.** Indicators of immune protection assay with recombinant *Bm*Trx-like protein (*Bm*TLP). His-tagged *Bm*TLP or PBS was used to immunize BALB/C mice (seven mice per group). After successful immunization, a challenge with 1 × 10^6^ *B. microti-*infected red blood cells was implemented. Body weight (A) and spleen weight (B) of the mice were detected and compared 15 days post infection. The results are representative of two independent experiments. Data indicate the mean ± standard deviation. *** indicates comparison between Blank and *Bm*TLP groups, Δ indicates comparison between *Bm*TLP and PBS groups, with Δ indicates p < 0.05 and *** indicates p < 0.0001.
